# Control of the Photo-Isomerization Mechanism in 3*H*-Naphthopyrans to Prevent Formation of Unwanted Long-Lived Photoproducts

**DOI:** 10.3390/ijms21217825

**Published:** 2020-10-22

**Authors:** Sabina Brazevic, Stanisław Nizinski, Michel Sliwa, Jiro Abe, Michał F. Rode, Gotard Burdzinski

**Affiliations:** 1 Faculty of Physics, Adam Mickiewicz University in Poznan, Uniwersytetu Poznanskiego 2, 61-614 Poznan, Poland; sabina.brazevic@amu.edu.pl (S.B.); stanislaw.nizinski@amu.edu.pl (S.N.); 2 Laboratoire de Spectroscopie pour les Interactions, la Réactivité et l’Environnement, LASIRE, CNRS, UMR 8516, Univ. Lille, 59000 Lille, France; michel.sliwa@univ-lille.fr; 3 Department of Chemistry, School of Science and Engineering, Aoyama Gakuin University, 5-10-1 Fuchinobe, Chuo-ku, Sagamihara, Kanagawa 252-5258, Japan; 4 Institute of Physics, Polish Academy of Sciences, Aleja Lotników 32/46, 02-668 Warsaw, Poland

**Keywords:** bicycle-pedal isomerization, naphthopyran, photochromism, photodynamics, photophysics, quantum chemical calculations, reaction mechanisms, time-resolved spectroscopy

## Abstract

In the photochromic reactions of 3*H*-naphthopyrans, two colored isomers TC (transoid-*cis*) and TT (transoid-*trans*) are formed. In terms of optimized photo-switchable materials, synthetic efforts are nowadays evolving toward developing 3*H*-naphthopyran derivatives that would not be able to photoproduce the long-living transoid-*trans*, TT, photoproduct. The substitution with a methoxy group at position 10 results in significant reduction of the TT isomer formation yield. The TC photophysics responsible for TT suppression were revealed here using a combination of multi-scale time resolved absorption UV-vis spectroscopy and ab initio calculations. The substitution changes the TC excited-state potential energy landscape, the *bicycle-pedal* isomerization path is favored over the rotation around a single double bond. The *bicycle-pedal* path is aborted in halfway to TT formation due to S_1_→S_0_ internal conversion populating back the TC species in the ground electronic state. This is validated by a shorter TC S_1_ state lifetime for methoxy derivative in comparison to that of the parent-unsubstituted compound (0.47 ± 0.05 ps vs. 0.87 ± 0.09 ps) in cyclohexane.

## 1. Introduction

**Scheme 1:**
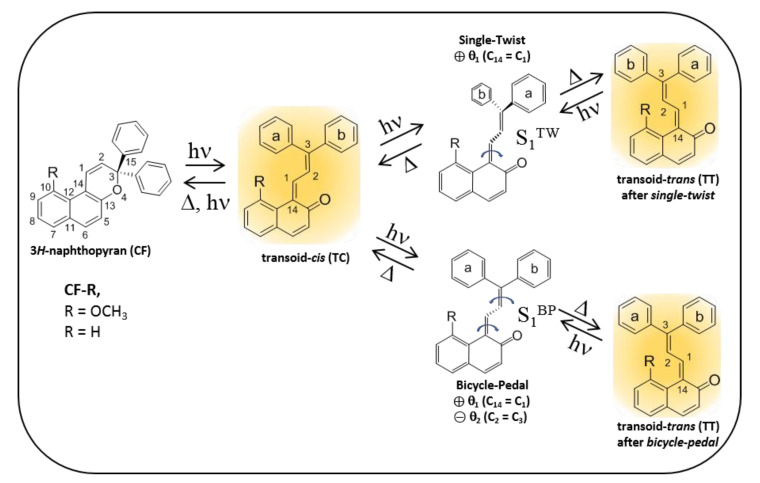
Two mechanistic pathways of the TC→TT photoisomerization process; the *single-twist* vs. *bicycle-pedal* motion. The presence of the methoxy group in 10-methoxy-CF (CF-OCH_3_) reduces the yield of the TT isomer formation [21]. θ_1_(C_14_ = C_1_) and θ_2_(C_2_ = C_3_) are two driving coordinates defined by the rotation about C_13_−C_14_=C_1_−C_2_ and C_1_−C_2_=C_3_−C_15_ dihedral angles, respectively.

Discovery of photochromic chromenes by Becker and Michl in 1966 initiated numerous studies related to their fundamental characterization and applications, especially as photochromic lenses [1]. One of the most frequently studied compounds belonging to this family is 3,3-diphenyl-3*H*-naphtho[2,1-*b*]pyran (see Scheme 1, CF-H, also known as 2,2-diphenyl-5,6-benzo(2*H*)chromene) [1,2,3,4,5,6,7,8,9,10,11,12,13,14,15,16,17,18,19,20,21].

The coloration process in this molecule—which is based on the photoinduced opening of the pyran ring in its closed form (CF)—is ultrafast as it takes place on a few ps time-scale leading to the transoid-*cis* TC form (see Scheme 1) [3,11,14,22,23,24,25]. In the next step transoid-*trans* TT form is produced by TC photoexcitation or is formed as a direct product of the pyran ring-opening [12,17]. A serious drawback of 3*H*-naphthopyran family is the long fading time of the transoid-*trans* TT isomer. While the TC form relaxes over tens of seconds, the persistence of the long-lived TT form over hours is a limiting factor for classical applications such as photochromic lenses, due to inconvenient slow fading response to UV light switch off. Thus, recent research has been focused on minimization of the TT formation yield in the photoreaction by designing new 3*H*-naphthopyran derivatives with appropriate substituents [21,22,26,27]. Aryl substituents in position 2 of 3*H*-naphthopyrans can exert both steric and electrostatic repulsion effects which have been suggested to suppress TT formation [22]. Exceptionally short TC lifetime, on the order of tens of microseconds in solution, make TC→TT photoisomerization inefficient upon stationary UV irradiation. However in this case, the photostationary formation yield of TC is also low. Keeping the TC lifetime of seconds and the suppression of the TT formation have been recently reported for 3*H*-naphthopyran with methoxy group substitution at position 10 (CF-OCH_3_, see Scheme 1) [21]. It has been proposed that the alkoxy group effectively reduces the formation of the transoid-*trans* form due to C−H···O intramolecular hydrogen bonding in the TC form [21]. The mechanism of TT suppression requires detailed studies of TC excited-state energy landscape.

Recently we have reinvestigated TT formation mechanism for unsubstituted 3*H*-naphthopyran (CF-H). We have shown that TC→TT photoisomerization competes with the other deactivation channels such as: S_1_→S_0_ internal conversion (major), S_1_→T_1_ intersystem crossing, S_1_ state fluorescence and allenyl-naphthol AP formation (hydrogen shift reaction) [25]. With this knowledge, we undertook comprehensive experimental and theoretical studies to explain why the presence of a methoxy group at position 10 of 3*H*-naphthopyran can so dramatically decrease the yield of TC→TT photoreaction. We apply multi-scale (from hundreds of femtoseconds to seconds) UV-vis time resolved absorption spectroscopy and quantum chemical calculations for 3*H*-naphthopyrans: CF-H and CF-OCH_3_. We have shown here for the first time two isomerization mechanisms TC→TT: (i) sole double bond isomerization with a *single-twist* competing with (ii) a *bicycle-pedal* isomerization (Scheme 1). The latter one is the dominant pathway for TC-OCH_3_ while the single-twist is the main one for TC-H. In the case of *bicycle-pedal* mechanism, isomerization is aborted in halfway to TT. Altogether, the *bicycle-pedal* has been shown a photostabilizing channel for TC form, thus this pathway plays a key role to effectively reduce TT concentration in the photoreaction.

## 2. Results and Discussion

### 2.1. The Effect of Methoxy Group Presence on the Ground-State Energy Landscape and the UV Absorption Spectrum

As far as the UV absorption spectrum is concerned, the calculations for CF-OCH_3_ molecule predict a very slight red-shift of the absorption maximum, in comparison to its position for the model chromene (CF-H), which in energy scale is not higher than 0.1 eV. This is in excellent agreement with experiment which gives the maximum of absorption at 361 nm in cyclohexane (3.44 eV) for CF-H [28] and 370 nm (3.35 eV) for CF-OCH_3_ (Figure S1). The observed red-shift is the effect of H substitution with a π-electron donating group, O−CH_3_, that decreases the excitation energy value of all the S_0_→S_n_(ππ*) electron transitions in CF. Such behavior has been observed earlier for methoxy substituted 3*H*-naphthopyrans (6-, 7- and 8-methoxy derivatives) [5,29].

**Figure 1:**
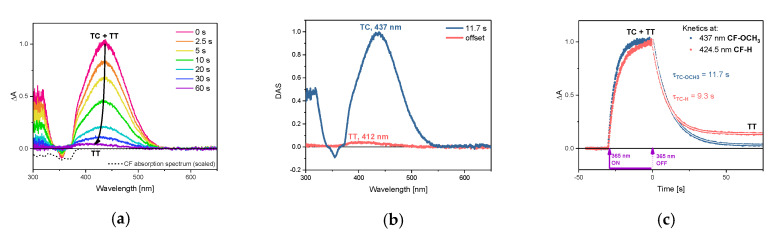
(**a**) UV-vis transient absorption spectra recorded for a solution of CF-OCH_3_ in cyclohexane (c ≈ 1 × 10^−4^ M) at 21 °C after turning off LED UV irradiation at 365 nm (5 mW/cm^2^); (**b**) Global analysis results in DAS (decay associated spectra) and two characteristic components: 11.7 s (TC lifetime) and the offset (TT); (**c**) Comparison of kinetics traces at 437 nm and 424.5 nm recorded for CF-OCH_3_ and CF-H respectively.

3*H*-Naphthopyrans typically produce both TC and TT isomers (see Scheme 1) under conditions of continuous UV irradiation tuned to the CF absorption band. These TC and TT isomers are structurally related through the rotation about the C_13_−C_14_=C_1_−C_2_ dihedral angle. The UV absorption spectra of TC and TT are then quite similar (see Tables S1 and S2) since the π-electron conjugation in those two forms is alike. Even though, in both forms the strongest S_0_→S_2_ transition is slightly red shifted for TC relative to its position for TT (Figure 1b). The methoxy group substitution does not change much the chromene’s ground-state energy landscape (calculated at the MP2/cc-pVDZ theory level) leaving the S_0_-state energy barriers almost unchanged [28]. Thus, similarly as in the H counterpart, TC-OCH_3_ and TT-OCH_3_ forms do not differ much energetically. The difference between the relative energies of the respective H and OCH_3_ derivatives is marginal, less than 0.02 eV, and both forms lie much above the closed-pyran form CF, app. by + 0.6 eV.

### 2.2. TT Formation, Single-vs. Two-Photon Mechanism

To unmix TT and TC contribution at the photo-stationary state under UV irradiation, the usual method is to follow thermal recovery to CF. Indeed after the decay of TC, only TT form remains. Figure 1a shows then time evolution of transient absorption spectra recorded for CF-OCH_3_ in cyclohexane after switching off UV irradiation (experimental set-up in Scheme S1). The initial absorption band corresponds to the mixture of TC (major) and TT (minor) species.

Figure 1b shows that the absorption maximum of TC-OCH_3_ occurs at 437 nm (2.84 eV), while that of TT-OCH_3_ isomer at 412 nm (3.01 eV), in cyclohexane (compare expt. vs theory in Table S1), corresponding to the spectral locations for H-derivative (427 nm, for TC-H, and 412 nm, for TT-H, Table S2). The TC lifetime is slightly longer for the methoxy form (11.7 s vs. 9.3 s in cyclohexane, see Table 1). A polar solvent as acetonitrile stabilizes the TC-OCH_3_ species (lifetime 17.0 s), indeed calculations show a substantial dipole moment of 4.2 D (Table S1). Figure 1c shows that TT isomer in cyclohexane is formed in smaller amount from CF-OCH_3_ than from CF-H precursor, in agreement with the data reported by Inagaki et al. [21]. On the basis of DAS amplitudes selected at the TT and TC maxima we estimate that CF-OCH_3_ in relation to CF-H described recently [25], produces four times less TT form under the same experimental conditions. Two parallel reaction mechanisms leading to TT isomer have been considered in literature [12,17]. One is a two-photon absorption process:

$$\mathrm{CF}\;\overset{365\;\mathrm{nm}}{\to }\mathrm{TC}\;\overset{365\;\mathrm{nm}}{\to }\mathrm{TT}$$

while the second one is a single photon excitation path:

$$\mathrm{CF}\;\overset{365\;\mathrm{nm}}{\to }\mathrm{TT}$$

**Figure 2:**
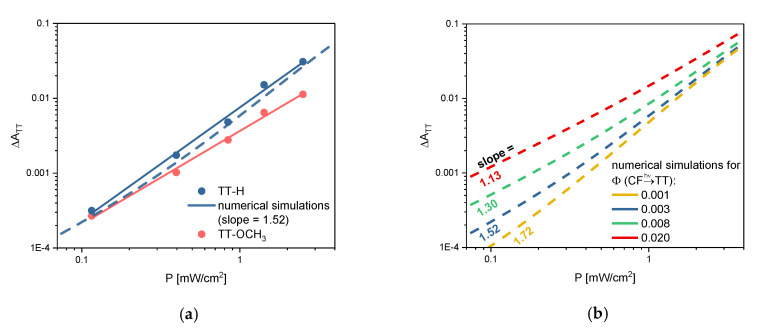
UV irradiation of CF in cyclohexane at 365 nm (3.40 eV) leads to TT population. (**a**) Plot of the TT absorption signal dependence on UV intensity on a log–log scale. The blue and red solid lines with slopes of 1.5 for TT-H and 1.2 for TT-OCH_3_, respectively, are the best linear fit to the observed data. (**b**) Numerical simulations for TT-H traced for various yields Φ of the direct single photon excitation path $\mathrm{CF}\;\overset{h\upsilon }{\to }\mathrm{TT}$.

Experiments performed for CF-H to characterize the influence of the UV irradiation power on the TT absorbance signal (Figure 2a) show the slope of this relation to be 1.5 in agreement with reported data [28]. The slope value close to 2 is expected for a purely biphotonic process. The discrepancy can be explained by a small contribution of the single-photon reaction, additionally to the main consecutive two-photon absorption reaction path. We performed numerical simulations (see SI for details) for CF-H in cyclohexane using photophysical properties determined recently [28] that quantify for the first time the yields of both processes. A quantum yield of 0.003 for the single-photon channel explains the experimental data (Figure 2b, the slope of 1.5). The single photon process leading to TT has intriguing nature, since it requires the initial ring-opening followed by significant changes in geometry, which contradicts the intuition. However, one can expect that upon ring-opening process TC form is initially generated in the vibrationally excited state. Such excess of vibrational energy can lead the hot TC molecule to overcome relatively high (~1.2 eV) S_0_-state energy barrier to form TT isomer.

The experimental slope determined for CF-OCH_3_ is closer to unity (1.2, Figure 2a), which indicates a greater contribution of the single photon excitation path in the TT-OCH_3_ formation (CF→TT) that correlates to a lower TT-OCH_3_ signal level. The reason for the decrease in the share of biphotonic channel (CF→TC→TT) is linked to the photo-dynamics of TC-OCH_3_ and needs thus to be determined precisely.

### 2.3. Photophysical Properties of TC-OCH_3_ in the Singlet Excited State

**Figure 3:**
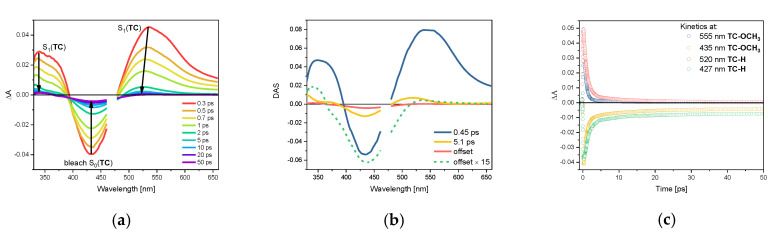
(**a**) Transient absorption UV-vis spectra recorded for TC-OCH_3_ in cyclohexane (c ≈ 3 × 10^−4^ M) upon excitation at 475 nm. (**b**) Global analysis shows decay associated spectra DAS with two time-constants 0.45 and 5.1 ps, and the offset. (**c**) Selected kinetic traces showing the TC S_1_ state decay and TC S_0_ repopulation. Data for TC-OCH_3_ and TC-H were collected in the same experimental conditions.

Ultrafast transient UV-vis absorption experiments were performed for TC-OCH_3_ in cyclohexane with photoexcitation at 475 nm (2.61 eV). In these experiments, a solution of CF-OCH_3_ was under a continuous LED UV irradiation at 365 nm (3.40 eV) to ensure a constant TC-OCH_3_ concentration. Although TT is also produced, its concentration is over 20 times lower than that of TC. Moreover the selected pump excitation wavelength at 475 nm (2.61 eV) favors TC excitation over TT on the basis of the respective absorption band locations (Figure 1b). Thus, the measured transient absorption spectra can be securely assigned to the sole excitation of TC-OCH_3_. Figure 3a shows the evolution of the transient absorption bands, which resemble the data reported for TC-H [25]. The initial positive transient absorption band peaking at 525 nm (2.36 eV) corresponds to TC-OCH_3_ in the singlet excited state (S_1_→S_n_), which is in agreement with theoretical calculations (Table S3). The band undergoes a substantial decay (88%) in the time window 0.3–2 ps which is concomitant with the recovery of the negative band peaking at 435 nm. The negative band corresponds to the depopulation band, i.e., depletion of the TC-OCH_3_ in the S_0_ state caused by laser pulse excitation at 475 nm (2.61 eV). The global analysis indicates two characteristic time-constants (see Figure 3b): 0.45 ps - related to the lifetime τ_S1_ of TC-OCH_3_ in the S_1_ state, and 5.1 ps corresponding to the vibrationally hot S_0_ species produced by S_1_→S_0_ internal conversion. The lifetime τ_S1_ can be also obtained from analysis of the band integral kinetics (0.47 ± 0.05 ps, Figure S2). The offset shows a weak positive band at 535 nm (2.32 eV) (see the offset × 15 in Figure 3b), which is assigned to the triplet excited state T_1_ produced by intersystem crossing S_1_→T_1_ as in the parent TC-H compound [25].

A significant shortening of singlet excited state lifetime for the methoxy TC derivative in comparison to the H-parent compound (0.47 vs. 0.87 ps) is observed in cyclohexane (Table 1).

Moreover, data comparison at 50 ps delay (Figure 3a) shows less pronounced S_0_ depopulation. Both observations can be explained by a more effective S_1_→S_0_ internal conversion channel in TC-OCH_3_ compound that rationalizes the decrease in TT formation in two photon process and then lower TT concentration in the photostationary state in comparison to those for the unsubstituted parent compound. The next step is to understand the reason for this increase in internal conversion rate and this requires the help of quantum chemical calculations.

### 2.4. Theoretical Modelling of TC→TT Photoisomerization Mechanism

In order to study the mechanism of the TC→TT photoisomerization reaction, it should be noted first that the molecular mechanism of rotation of the whole rotor unit vs. naphthalenone skeleton may be realized along the two different pathways (variations). They can be classified as *single-twist* or *bicycle-pedal* motion [30,31,32,33,34].

**Figure 4:**
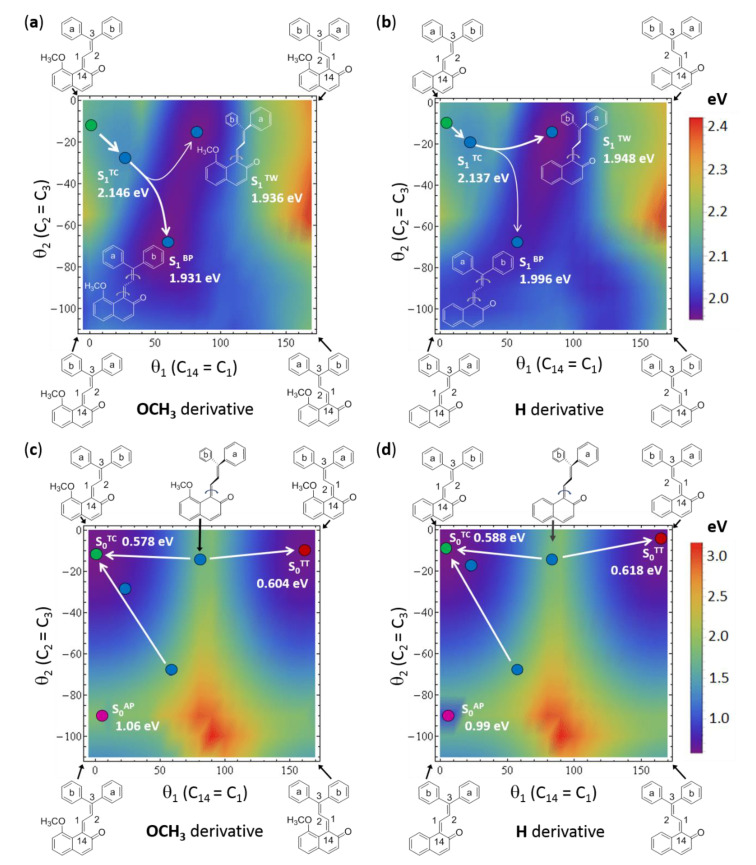
Minimum-potential-energy surface of the lowest excited electronic state (upper row, (**a**,**b**)) and the ground state (lower row, (**c**,**d**)) of H (right column) and its methoxy derivative (left column) plotted as a function of θ_1_(C_14_ = C_1_) and θ_2_(C_2_ = C_3_) coordinates. The energy values at selected geometries are given in eV. Green, red and magenta circles represent the FC region of the ground-state S_0_^TC^, S_0_^TT^, and S_0_^AP^ local minima, respectively. Blue circles represent various types of excited-state minima: S_1_^TC^—the minimum initially populated after S_0_^TC^ photoexcitation, S_1_^BP^—achieved through the *bicycle-pedal* motion, and S_1_^TW^—reached by *single-twist* motion mechanism. Bold white arrows indicate the expected downhill motion of the wavepacket following the photoexcitation of TC form in its FC region. The results were obtained with the aid of the ADC(2)/cc-pVDZ method for the excited state and with the MP2/cc-pVDZ for the ground state.

To visualize these two variations of the TC→TT photoisomerization process, the excited state (S_1_) and ground-electronic state (S_0_) two-dimensional minimum potential energy surfaces (S_1_-PES, and S_0_-PES, respectively) were constructed to compare the photoisomerization mechanism between the OCH_3_ and H derivatives (see Figure 4). The molecule first evolves along the S_1_-PES toward the region where the S_1_→S_0_ internal conversion process takes place (Figure 4a,b, for OCH_3_ and H derivatives). Further evolution of the molecule follows the S_0_-state gradient toward the stable minimum (Figure 4c,d). The PES minima of the ground state, S_0_, and of the lowest excited state, S_1_, were calculated using the MP2/cc-pVDZ and ADC(2)/cc-pVDZ methods, respectively. Each energy point at PES was obtained by optimization of the geometry of a given molecule imposing two constraints for driving coordinates: θ_1_(C_14_ = C_1_) and θ_2_(C_2_ = C_3_), separately, in a given electronic state. These two driving coordinates were frozen while all the remaining 3N-8 coordinates were optimized for each point in given electronic state. Thus, each PES is spread over the two driving coordinates: θ_1_(C_14_ = C_1_) and θ_2_(C_2_ = C_3_) (see Scheme 1, for definition) defined as the dihedral angles describing rotation about the respective double bond. Note also that the two phenyl rings in the rotor unit were distinguished and marked with letters: “a” and “b” in the molecular structure (see Scheme 1 and Figure 4) to discriminate between the *single-twist* and *bicycle-pedal* variant motions. In the excited state, the photoisomerization can be realized as the *single-twist* described as the rotation of the rotor unit vs. naphthalenone skeleton about the sole C_14_ = C_1_ bond and could be observed along the direction parallel to the θ_1_(C_14_ = C_1_)-axes in Figure 4a,b, for OCH_3_ and H derivatives, respectively. The isomerization process, however, can be realized alternatively – as a *bicycle-pedal* motion – in which the concerted rotation about the two double bonds: θ_1_(C_14_ = C_1_) and θ_2_(C_2_ = C_3_) takes place simultaneously. This movement can be seen as the two benzene rings: a and b moving in direction parallel to the naphthalenone moiety plane. The *bicycle-pedal* motion can be observed along the direction close to the diagonal of Figure 4a,b. It links upper-left and bottom-right corners of the respective S_1_-PES so that the condition Δθ_1_ = −Δθ_2_ is approximately fulfilled.

**Figure 5:**
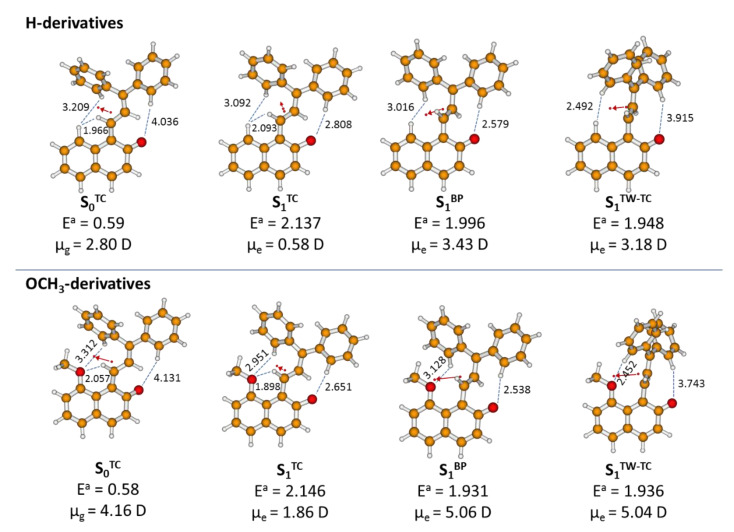
Considered ground and excited-state equilibrium geometries potentially involved in the TC→TT photoisomerization process of H (upper row) and OCH_3_ (lower row) derivatives optimized at the MP2/cc-pVDZ(S_0_) and ADC(2)/cc-pVDZ theory level. The corresponding intramolecular bond distances (in Å), adiabatic energy (E^a^ in eV), the dipole moment of the ground (μ_g_, in D) and the excited-state form (μ_e_, in D) are given.

Photoexcitation of TC isomer in its Franck-Condon region populates the ππ* excited state. Next, the excited-state relaxation proceeds in a barrierless fashion from the TC Franck-Condon region toward the relaxed excited-state minimum, S_1_(TC) form. As shown for the unsubstituted molecule [25], this relaxation process is accompanied by elongation of the C_13_ = O_4_ carbonyl double bond which eventually becomes single and the excited state gains the nπ* character. This mechanism resembles that discovered for DNA bases in which the nπ* state is responsible for driving the system in the region of the conical intersection with the ground electronic state CI(nπ*/S_0_) [35,36]. Thus, the populated excited-state minimum S_1_(TC) is observed in the ultrafast experiment with transient absorption detection (Figure 3). This molecule evolves further along a few deactivation channels [25], including the one that drives the molecule toward the TT photoproduct. A detail analysis of the mechanism of this process was possible thanks to the use of the calculated PES. The relevant species obtained as a result of the excited-state geometry evolution are shown in Figure 5 and Table 2.

We start our analysis of the process with the ground-state S_0_^TC^ geometry whose position is marked with the green dot in the upper-left corner of Figure 4a,b, respectively, for methoxy and H derivatives. The single-photon excitation of S_0_^TC^ form of each molecule in a barrierless manner populates the excited-state S_1_^TC^ minimum. The first difference between the molecules occurs in the position of the S_1_^TC^ minimum on the corresponding S_1_-PES (blue dot in the upper left corner of Figure 4a,b).

During the initial S_1_-state relaxation process of TC, both double bonds (θ_1_, θ_2_) become twisted from their initial Franck-Condon region values (1.8°, −11.7°), for S_0_^TC^(OCH_3_), down to (25.5°, −27°), for S_1_^TC^(OCH_3_) minimum; and from the initial values (2.5°, −9.5°), for S_0_^TC^(H), down to (22.5°, −18.7°), for S_1_^TC^(H) minimum (see Table 2). In case of the H derivative, the S_1_^TC^ geometry points toward the *single-twist* motion path along the direction parallel to the θ_1_(C_14_ = C_1_)-axis (θ_1_ = 22.5°, θ_2_ = −18.7°).

However, the S_1_^TC^(OCH_3_) geometry (θ_1_ = 25.5°, θ_2_ = −27.0°) lies much closer to the diagonal of the S_1_-PES, where the θ_1_ = −θ_2_ *bicycle-pedal* condition is fulfilled. The S_1_^TC^ form becomes an intermediate, whose geometry may determine further evolution of the molecule in the electronic excited state toward TT along the *single-twist* or *bicycle-pedal* pathway. These two mechanism variations populate a given type of the excited-state minimum: S_1_^BP^, for *bicycle-pedal*, or S_1_^TW^, for *single-twist* motion, respectively, shown in Figure 5.

For OCH_3_ derivative (Figure 4a), the violet region forms a double minimum valley linking the two almost isoenergetic excited-state minimum geometries (blue dots): S_1_^BP^ and S_1_^TW^ separated by a very low energy barrier. S_1_^TW^ [θ_1_ = 82.6°, θ_2_ = −13.7°] is located in the midpoint between the S_0_^TC^ and S_0_^TT^ geometries (green and red circles) illustrating t*wisting* motion of the molecule. The second minimum, S_1_^BP^ [θ_1_ = 58.3°, θ_2_ = −69.1°], is located in the region close to the diagonal of Figure 4a suggesting that the *bicycle-pedal* mechanism should prevail over the *twisting* motion in the OCH_3_ molecule, for the energy reasons.

The S_1_^BP^ and S_1_^TW^ minima were also determined for the unsubstituted parent compound. In this case, the S_1_^TW^ excited-state global minimum [θ_1_ = 84.7°, θ_2_ = −14.9°] is more energetically favored. It is by 0.05 eV more stable than that of the corresponding S_1_^BP^ form [θ_1_ = 59.2°, θ_2_ = −68.5°]. As a consequence, the position of the deeper twist-type S_1_^TW^ global minimum on S_1_-PES suggests that the *twisting* motion prevails over the *bicycle-pedal*, for the model of unsubstituted compound. Furthermore in agreement with recently reported experiments [25] we can also rationalize that the use of polar solvent is another way to control the equilibrium between *bicycle-pedal* and *single-twist* motion leading to the suppression of the TT formation in the H derivative. Indeed, in a polar solvent, the *bicycle-pedal* pathway will be favored over *single-twist* motion since the former path leads to a more polar intermediate S_1_^BP^ (Figure 5), which likely produces back TC form after S_1_→S_0_ internal conversion. Moreover, in a polar solvent such as acetonitrile, the lifetime τ_S1_ of TC is shorter in comparison to that in a non-polar cyclohexane (0.27 ps vs. 0.47 ps for OCH_3_ derivative and 0.31 ps vs. 0.87 ps for H derivative, see Table 1) [25].

Even though after photoexcitation of TC-OCH_3_ the excited-state gradient pulls the molecule toward the S_1_^BP^ region, the S_1_-state PES still indicates the existence of a low-lying flat energy valley reaching toward the region located around θ_2_ = −90° (blue region on the lower-left part of Figure 4c,d). This is the region illustrating sole *single-twist* motion around the C_2_ = C_3_ double bond of the H and OCH_3_ derivatives. During this alternative *twist* motion, the proton attached to the C_2_ carbon atom in allene chain is found in vicinity of the carbonyl oxygen atom, O_4_, that pulls the proton to form a stable AP minimum. The ground-state energy surface S_0_-PES of both molecules indicates the existence of a high-energy ground-state equilibrium naphthalenol AP form (see the structure in Table S2). Depopulation of the excited state in this region may generate the AP form in the electronic ground state. The formation of the AP form has been detected in NMR studies for the TC-H molecule [8].

### 2.5. Role of the Methoxy Group

The π-electron-donating character of the methoxy group has been studied recently in both, ground- [37] and excited state [38] of monosubstituted benzenes. The electron donating effect of the methoxy group is even greater in the excited S_1_ state than in the S_0_ state. The presence of a methoxy group in the species shown in Figure 5 results in a greater dipole moment of either the ground- or excited state due to a shift of electron-density from the methoxy group toward the carbonyl oxygen atom, O_4_. The O_4_ oxygen atom attracts phenyl b ring more in OCH_3_ than in H derivative. It results in energetic stabilization of the corresponding excited-state species, S_1_^BP^ and S_1_^TW^. In OCH_3_ derivative, shortening of the C=O_4_···H distance stabilizes the *bicycle-pedal* type excited-state species S_1_^BP^ vs. the *single-twist* one. Thus, we claim here that the role of the methoxy group is mostly to shift electron density toward the carbonyl oxygen atom which attracts the whole rotor unit more strongly than in unsubstituted derivative. This strengthen attraction results in a slight tilt of the rotor toward the carbonyl group in all the structures in the singlet excited state (C_10_-C_1_ and C_10_-C_2_ distances increase by 0.1 Å). The electronic effect is affecting the photophysics of the process and is dominating over the steric effect caused by the incorporation of the relatively small methoxy group into the molecule. This thesis is supported by experimental finding that 10-bromo substituted compound photoproduces a similar TT yield as the H derivative [21]. However the steric effect with more bulky substituents needs further investigations.

The molecular system in the excited S_1_ state can deactivate to the electronic ground state, S_0_, through S_1_→S_0_ internal conversion. Thus, the ground state S_0_-PES was constructed for both molecules (see Figure 4a,b, for H and OCH_3_, respectively). The height of the S_0_-state energy barrier precludes the TC→TT photoisomerization process to be thermally activated.

As one can see, the blue dot representing the geometry of *twisting* type excited-state S_1_^TW^ minimum is located close to the top of the green region representing the S_0_-state energy barrier for both molecules. The system after S_1_-state deactivation follows the S_0_-state energy gradient and eventually gains toward the TT photoproduct (S_0_^TT^), or goes back to TC form (S_0_^TC^).

In contrast to the twisting motion, the blue dot representing the *bicycle-pedal* type S_1_^BP^ minimum is positioned on the left side of the S_0_-state energy barrier. The deactivation of S_1_^BP^ favors return of the system toward the ground-state TC form, since once being in the ground state, the molecule meets a high barrier for the TC→TT isomerization and the ground-state energy gradient pulls it back to TC isomer. Thus, the channel path populating the S_1_^BP^ minimum may result in stopping the photoisomerization process. The *bicycle-pedal* motion in the S_1_-state prevails over the *single-twist* for OCH_3_ derivative, thus a lower TC→TT isomerization quantum yield for this molecule can be expected, which is in agreement with experimental finding.

## 3. Materials and Methods

### 3.1. Materials

CF-OCH_3_ (10-methoxy-3,3-diphenyl-3*H*-benzo[*f*]chromene) was synthesized and purified accordingly to previously described procedures [21]. CF-H (3,3-diphenyl-3*H*-benzo[*f*]chromene) was purchased from TCI (Springfield, Virginia, USA).

### 3.2. Transient UV-Vis Absorption Spectra Over Seconds

UV LED (λ_exc_ = 365 nm, M365LP1, Thorlabs, Newton, New Jersey, USA) was used to perform photochromic reaction in a 1 cm × 1 cm fused silica cuvette placed in a temperature-controlled cuvette holder (Flash 300, Quantum Northwest, Liberty Lake, Washington, USA) with stirring (see Scheme S1). The volume of the solution was about 1.5 mL. Changes in UV-vis absorption spectra over seconds were recorded by a FLAME-T-VIS-NIR-ES USB spectrometer (6 s^−1^ sampling rate, Ocean Optics, Largo, Florida, USA). White-light of 150 W xenon lamp (Applied Photophysics, Leatherhead, Surrey, United Kingdom) with intensity reduced to a small level was used as a probing beam. 150 ms were used for accumulation of each white-light continuum spectrum. An average of 60 initial spectra before sample UV-irradiation was used to calculate $I_{0}\left(\lambda \right)$ spectrum, the next spectra I(λ) at subsequent times were measured upon or after UV-irradiation to determine the transient absorption spectra accordingly to formula: $\Delta A\left(\lambda \right)=\log\frac{I_{0}\left(\lambda \right)}{I\left(\lambda \right)}$.

In order to study influence of UV irradiation power on TT formation a V-550 spectrophotometer (Jasco, Hachioji-shi, Tokyo) equipped with the mentioned above temperature-controlled cuvette holder, stirring and UV LED source was used.

### 3.3. Transient UV-Vis Absorption Over Picoseconds

Femtosecond UV-vis transient absorption spectra were obtained using a commercially available system (Ultrafast Systems, Helios, Sarasota, Florida, USA) [39]. Photostationary state of TC was obtained by continuous UV irradiation (5 mW/cm^2^) at 365 nm of CF solution in a quartz cell 2 mm thick with stirring.

The instrument response function (IRF) was about 200 fs (FWHM) estimated from a stimulated Raman scattering observed in pure solvent. The energy of laser pulses at 475 nm was 1 μJ. The data set was composed of a series of 280 spectra acquired for pump-probe delay ranging from -1 to 50 ps. Each transient absorption ΔA spectrum was calculated from 500 pairs of probe (pumpON) and reference (pumpOFF) spectra, it corresponded to 1 s of acquisition time (see Scheme S2). The obtained transient absorption spectra were analyzed using the global fitting procedure (ASUFIT program), satisfactory fits were obtained with a double-exponential function. Convolution with the instrument response function was included into the fitting procedure.

The accuracies of the obtained time-constants derived from analysis of transient absorption results were following: ± 5% (UV-vis data in time window over tens of seconds) and ± 10% (ultrafast UV-vis data).

### 3.4. Computational Details

The equilibrium geometries of the OCH_3_ and H derivatives of 3*H*-naphthopyran and its isomers in their closed-shell ground state (S_0_) were obtained with the MP2 method [40] without imposing any symmetry constrains. The energy of the most stable form CF is the reference energy for higher energy structures. The excited-state (S_1_) equilibrium geometries were determined with the second-order algebraic diagrammatic construction ADC(2) method [41,42,43]. The correlation-consistent valence double zeta basis set with polarization functions on all atoms (cc-pVDZ) was used in these calculations as well as in potential energy surfaces [44]. The vertical excitation energies and response properties of the lowest excited singlet states were calculated using the ADC(2) and CC2 methods [45,46]. The aug-cc-pVDZ basis set augmented with the diffuse functions was also used to compute vertical excitation energies of the OCH_3_ and H molecular system. All calculations were performed using TURBOMOLE program package [47].

## 4. Conclusions

The experiments in cyclohexane show TT photoreaction yield four times lower for methoxy derivative than for the parent unsubstituted compound. Ultrafast experiments demonstrated that the excited state lifetime τ_S1_ of TC-OCH_3_ derivative is shorter (0.47 ± 0.05 ps) than that of TC-H (0.87 ± 0.09 ps) due to more effective radiationless S_1_→S_0_ internal conversion channel, populating back the TC form. Theoretical studies show two possible variants of photoisomerization reaction: (i) *single-twist* and (ii) *bicycle-pedal* motions. As demonstrated by the calculations, in photoexcited TC-OCH_3_ the *bicycle-pedal* motion is energetically more favored than the *single-twist*. The *bicycle-pedal* motion leads to a minimum in S_1_^BP^ which is located in a close proximity of S_0_-PES facilitating S_1_→S_0_ internal conversion, which through further structural evolution leads toward the relaxed TC minimum. The *bicycle-pedal* channel is less probable in H counterpart, which favors population of the single-twisted form S_1_^TW^. The excited-state decay of S_1_^TW^ leads to the ground state at the geometry close-lying to the S_0_-state isomerization barrier, thus the energy gradient can drive the molecule toward TT or back to the TC minimum. Our studies have shown that the choice of appropriate substituent or polar solvent can favor the *bicycle-pedal* TC→TT isomerization path, which can be aborted leading to the decrease in TT formation yield. Altogether, the *bicycle-pedal* path is seen as photostabilizing channel for TC form and opens the way to design new efficient naphthopyrans free from TT formation.

## Figures and Tables

**Table 1:** Photophysical properties of TC in solution: S_0_ and S_1_ absorption band maxima, lifetime τ_S0_ and excited state lifetime τ_S1._

Compound	Solvent	S_0_(TC) λ_abs_^max^	τ_S0_, s	S_1_(TC) λ_abs_^max^	τ_S1_, ps
TC-OCH_3_	cyclohexane	437 nm	11.7 ^1^	525 nm ^3^	0.47 ^2^
	acetonitrile	447 nm	17.0 ^1^	545 nm	0.27 ^2^
TC-H	cyclohexane	427 nm	9.3 ^1^	520 nm ^3^	0.87 ^2^
	acetonitrile	430 nm	8.6 ^1^	513 nm	0.31 ^2^

Temperature: ^1^ 21 °C; Accuracy: ^1^ ± 5%; ^2^ ± 10%; τ_S1_ retrieved from band integral kinetics; ^3^ in agreement with calculations (Tables S3 and S4, respectively).

**Table 2:** Comparison of geometric parameters (bond lengths, in Å, dihedral angles), adiabatic energies (*E_a_,* in eV) for the excited state (S_1_) geometries optimized at the ADC(2)/cc-pVDZ level of theory. σ and τ denote C_14_−C_1_−C_2_−C_3_ and C_5_−C_13_−C_14_−C_1_ dihedral angles, respectively.

S_0_- or S_1_-State Form	R_1_ C_3_−O_4_ [Å]	θ_1_ C_13_−C_14_=C_1_−C_2_ [°]	θ_2_ C_1_−C_2_=C_3_−C_15_ [°]	σ [°]	τ [°]	R_2_ C_13_−O_4_ [Å]	R_D_ C_1_−C_14_ [Å]	R_S_ C_1_−C_2_ [Å]	R_E_ C_2_−C_3_ [Å]	E_a_ [eV]
H-derivative										
S_0_^TC^	4.165	2.5	−9.5	173.5	160.3	1.239	1.386	1.437	1.384	0.59
S_1_^TC^	3.921	22.5	−18.7	−174.8	174.2	1.399	1.478	1.371	1.440	2.14
S_1_^TW^	4.433	84.7	−14.9	−170.0	180.0	1.266	1.474	1.366	1.445	1.95
S_1_^BP^	4.243	59.2	−68.5	−176.5	176.6	1.266	1.482	1.344	1.492	2.00
OCH_3_-derivative										
S_0_^TC^	4.086	1.8	−11.7	173.6	153.3	1.239	1.389	1.439	1.383	0.58
S_1_^TC^	3.818	25.5	−27.0	−174.9	172.8	1.390	1.483	1.364	1.450	2.15
S_1_^BP^	4.170	58.3	−69.1	−177.8	178.7	1.263	1.483	1.342	1.492	1.93
S_1_^TW^	4.308	82.6	−13.7	−177.2	177.3	1.260	1.476	1.366	1.439	1.94

## Supplementary Materials

Supplementary materials can be found at https://www.mdpi.com/1422-0067/21/21/7825/s1.
