# Supplementary material for: Control of the Photo-Isomerization Mechanism in 3H-Naphthopyrans to Prevent Formation of Unwanted Long-Lived Photoproducts
Source: Int J Mol Sci. 2020 Oct 22;21(21):7825. doi: 10.3390/ijms21217825 (PMC7659934; doi:10.3390/ijms21217825)
Supplement: Supplementary file 1 [file ijms-21-07825-s001.pdf]

Supplementary Information

Control of the Photo-isomerization Mechanism in 3*H*-Naphthopyrans to Prevent Formation of Unwanted Long Lived Photoproduct

Sabina Brazevic [1], Stanisław Nizinski [1], Michel Sliwa [2], Jiro Abe [3,*], Michał F. Rode [4,*] and Gotard Burdzinski [1,*]

[1] Faculty of Physics, Adam Mickiewicz University in Poznan, Uniwersytetu Poznanskiego 2, 61-614 Poznan, Poland
[2] Laboratoire de Spectroscopie pour les Interactions, la Réactivité et l'Environnement, Univ. Lille , CNRS, UMR 8516, LASIRE, F-59000 Lille, France
[3] Department of Chemistry, School of Science and Engineering, Aoyama Gakuin University, 5-10-1 Fuchinobe, Chuo-ku, Sagamihara, Kanagawa 252-5258, Japan
[4] Institute of Physics, Polish Academy of Sciences, Aleja Lotników 32/46, 02-668 Warsaw, Poland

Table of Contents

Scheme S1 .... **S2**
Scheme S2 .... **S2**
Figure S1 .... **S3**
Figure S2. .... **S3**
Numerical simulations - TT formation **S4**
Table S1 .... **S5**
Table S2 .... **S5**
Table S3 .... **S6**
Table S4 .... **S6**
References **S7**

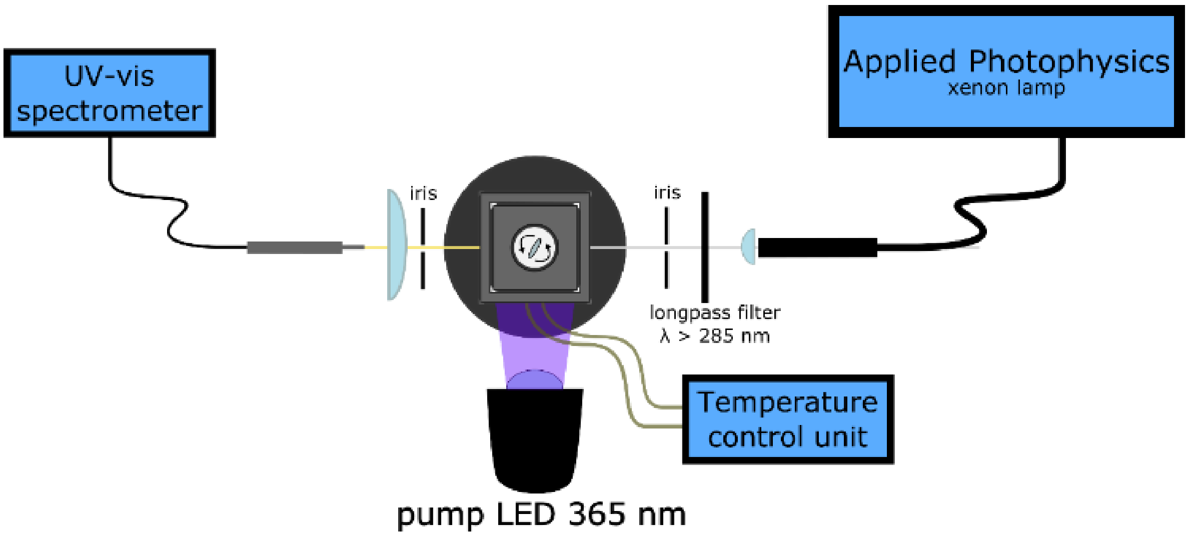

Scheme S1. Set-up for measuring UV-Vis transient absorption spectra over seconds.

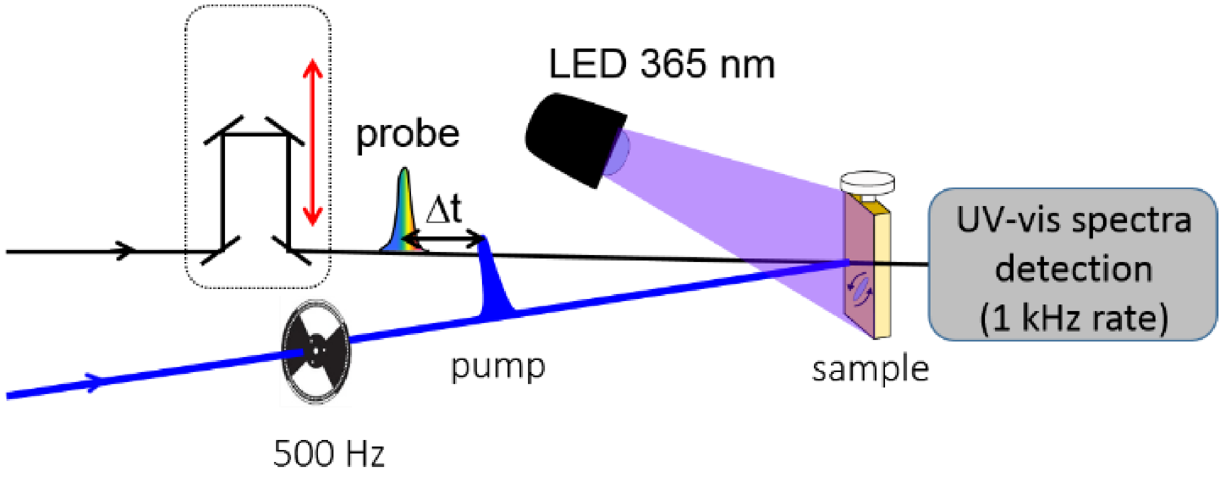

Scheme S2. Set-up for measuring UV-vis transient absorption spectra over picoseconds.

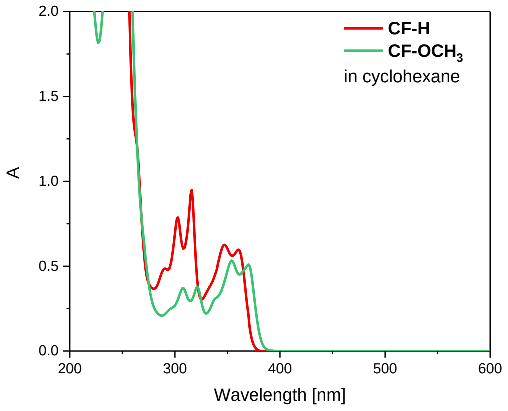

Figure S1. Stationary UV-vis absorption spectra of **CF-H** and **CF-OCH₃** in cyclohexane.

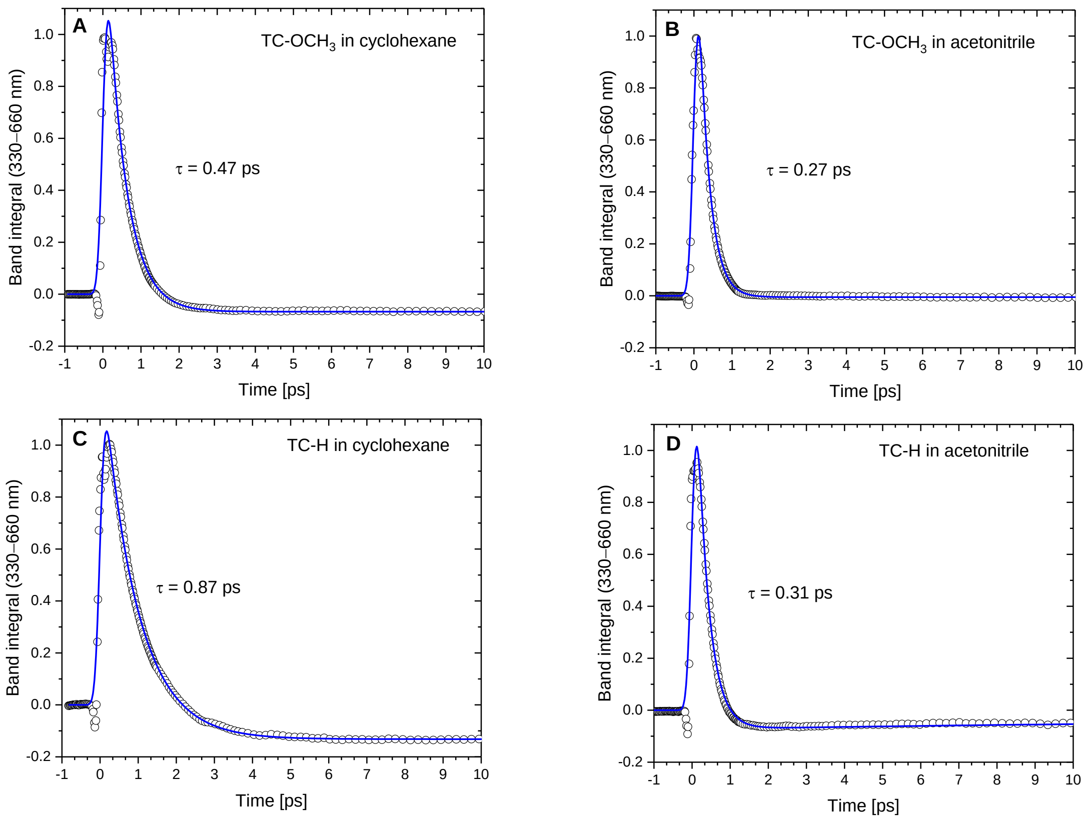

Figure S2. Calculated band integral kinetics over 330–660 nm, according to the equation $BI(t) = \int_{330}^{660} \Delta A(t,\lambda)\frac{d\lambda}{\lambda}$, for transient absorption spectra measured for: **TC-OCH$_3$** in cyclohexane (A) and acetonitrile (B), **TC-H** in cyclohexane (C) and acetonitrile (D). To retrieve the **TC** S_1 excited state lifetimes τ data were fitted with a single-exponential function convoluted with IRF (200 fs). Note that analysis of the band integral kinetics is known to minimize contributions from vibrational relaxation and solvation [1].

Numerical simulations - TT formation

To build simulated log-log plots, we used NDSolve function in Mathematica software, which allowed us to solve numerically set of differential equations, which describe observed reactions.

Initial conditions:

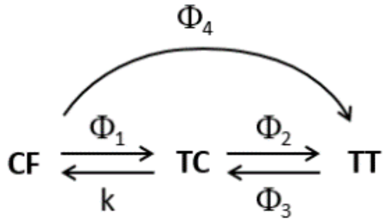

$\varepsilon_{CF}[365] = 3389$ L mol^{-1} cm^{-1}
$\varepsilon_{CF}[450] = 0$ L mol^{-1} cm^{-1}
$\varepsilon_{TC}[365] = \varepsilon_{CF}[365]$
$\varepsilon_{TC}[450] = 16154$ L mol^{-1} cm^{-1}
$\varepsilon_{TT}[365] = \varepsilon_{CF}[365]$
$\varepsilon_{TT}[450] = 11850$ L mol^{-1} cm^{-1}
$\varphi_1 = 0.745$
$\varphi_2 = 0.1$
$\varphi_3 = 0.107$
k = 1/11.7 s^{-1}

Differential equations to solve:

$$\mathrm{CF}'[t] = -\phi_1 F[t] I_0 \epsilon_{\mathrm{CF}}[365] l_i \mathrm{CF}[t] + TC[t]\, k - \phi_4\, F[t] I_0\, \epsilon_{\mathrm{CF}}[365]\, l_i\, CF[t]\,,$$
$$\mathrm{TC}'[t] = \phi_1 F[t] I_0 \epsilon_{\mathrm{CF}}[365] l_i \mathrm{CF}[t] - \phi_2 F[t] I_0 \epsilon_{\mathrm{TC}}[365] l_i \mathrm{TC}[t] - \mathrm{TC}[t]\,\mathrm{k} + \phi_3 \mathrm{F}[\mathrm{t}] I_0 \epsilon_{\mathrm{TT}}[365] l_i \mathrm{TT}[\mathrm{t}],$$
$$\mathrm{TT}'[t] = \phi_2 F[t] I_0 \epsilon_{\mathrm{TC}}[365] l_i \mathrm{TC}[t] - \phi_3\, F[t] I_0\, \epsilon_{\mathrm{TT}}[365]\, l_i\, TT[t] + \phi_4\, F[t] I_0\, \epsilon_{\mathrm{CF}}[365]\, l_i\, CF[t],$$

where CF[t], TC[t], TT[t] are concentrations, l_i is irradiation optical path length, l_p is probe optical path length, $F[\mathrm{t}] = \frac{1-\exp[-2.3A_T[t]]}{A_T[t]}$ is photokinetic factor, $A_T[t]$ is total absorbance at irradiation wavelength. These equations take into account two stages of two photon **CF→TT** photoconversion (ϕ_1 and ϕ_2), as well as one photon **CF→TT** photoconversion (ϕ_4) and **TT→TC** back reaction (ϕ_3). Rate constants of thermal reactions were determined separately by fitting decay kinetics measured in the dark. All quantum yields, except ϕ_4 have been determined experimentally by fitting experimental results using our kinetic model. Initial absorbance was experimentally determined using stationary spectrometer for non-irradiated sample. Equations were solved for each irradiation intensity from grid generated to cover entire range of irradiation intensities used in experiments, and for given ϕ_4, then $\varepsilon_{\mathrm{TT}}[450]\ l_p\ TT[30s]$ was plotted versus I_0 expressed in mW/cm^2.

Table S1. Vertical excitation energy (ΔE^{VE}, in eV and λ_{abs}, in nm calculated *vs.* experimental), oscillator strength (*f*), and dipole moment (μ_e, in Debye) of the lowest singlet states for the equilibrium forms of the OCH_3-derivative calculated with the CC2/aug-cc-pVDZ method at the ground state geometries optimized at the MP2/cc-pVDZ theory level. Italic indicates the ground state values.

S_0 form		ΔE^{VE} calc.	λ_{abs} calc.	*f* calc.	μ_e calc.	ΔE^{VE} expt.	λ_{abs} expt.
**CF-OH3** 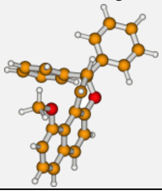	S_0	*0.0*			*2.2* [b]		
	$S_0\rightarrow S_1(\pi\pi^*)$	3.58	347	0.111	3.3	3.35 eV	370 nm
	$S_0\rightarrow S_2(\pi\pi^*)$	4.16	298	0.029	4.0		
	$S_0\rightarrow S_3(n\pi^*)$	4.57	272	0.002	7.0		
	$S_0\rightarrow S_4(\pi\pi^*)$	4.73	262	0.068	4.5		
	$S_0\rightarrow S_5(n\pi^*)$	4.83	257	0.004	1.2		
	$S_0\rightarrow S_6(\pi\pi^*)$	4.85	256	0.135	1.7		
**TC-OCH3** 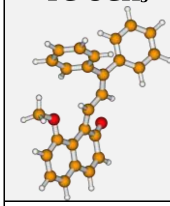	S_0	*0.58* [a]			*4.2* [b]		
	$S_0\rightarrow S_1(\pi\pi^*)$	2.75	451	0.132	3.0		
	$S_0\rightarrow S_2(\pi\pi^*)$	2.95	421	0.549	5.8	2.84 eV	437 nm
	$S_0\rightarrow S_3(\pi\pi^*)$	3.53	352	0.067	10.3		
	$S_0\rightarrow S_4(\pi\pi^*)$	4.02	309	0.067	6.1		
	$S_0\rightarrow S_5(\pi\pi^*)$	4.19	296	0.242	4.8		
	$S_0\rightarrow S_6(\pi\pi^*)$	4.44	280	0.014	5.8		
**TT-OCH3** 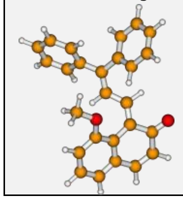	S_0	*0.60* [a]			*4.5* [b]		
	$S_0\rightarrow S_1(\pi\pi^*)$	2.78	446	0.117	2.8		
	$S_0\rightarrow S_2(\pi\pi^*)$	3.12	398	0.439	7.0	3.01 eV	412 nm
	$S_0\rightarrow S_3(\pi\pi^*)$	3.50	355	0.113	8.6		
	$S_0\rightarrow S_4(\pi\pi^*)$	3.89	319	0.024	6.0		
	$S_0\rightarrow S_5(\pi\pi^*)$	4.22	294	0.126	4.5		
	$S_0\rightarrow S_6(\pi\pi^*)$	4.52	275	0.021	7.8		

[a] adiabatic energy of the given form (*Ea*, in eV) relative to the ground-state closed form, **CF**, calculated at the MP2/cc-pVDZ theory level. [b] dipole moment calculated at the MP2/cc-pVDZ theory level.

Table S2. Vertical excitation energy (ΔE^{VE}, in eV and λ_{abs}, in nm calculated *vs.* experimental), oscillator strength (*f*), and dipole moment (μ_e, in Debye) of the lowest singlet states for the equilibrium forms of the **H**-derivative calculated with the **CC2/aug-cc-pVDZ** method at the ground state geometries optimized at the **MP2/cc-pVDZ** theory level. Italic indicates the ground state values.

S_0 form		ΔE^{VE} calc.	λ_{abs} calc.	*f* calc.	μ_e calc.	ΔE^{VE} expt.	λ_{abs} expt.
**CF-H** 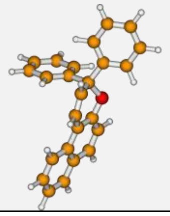	S_0	*0.0*			*0.9* [b]		
	$S_0\rightarrow S_1(\pi\pi^*)$	3.70	335	0.081	1.0	3.47 eV	361 nm
	$S_0\rightarrow S_2(\pi\pi^*)$	4.23	293	0.084	1.7		
	$S_0\rightarrow S_3(n\pi^*)$	4.60	270	0.004	3.3		
	$S_0\rightarrow S_4(\pi\pi^*)$	4.84	256	0.007	7.1		
	$S_0\rightarrow S_5(\pi\pi^*)$	4.97	250	0.067	1.1		
	$S_0\rightarrow S_6(\pi\pi^*)$	4.99	249	0.234	1.1		
**TC-H**	S_0	*0.59* [a]			*2.8* [b]		
	$S_0\rightarrow S_1(n\pi^*)$	2.71	458	0.031	1.3		
	$S_0\rightarrow S_2(\pi\pi^*)$	2.97	418	0.714	5.5	2.91 eV	427 nm
	$S_0\rightarrow S_3(\pi\pi^*)$	3.52	353	0.091	8.4		
	$S_0\rightarrow S_4(\pi\pi^*)$	4.15	299	0.143	5.5		
	$S_0\rightarrow S_5(\pi\pi^*)$	4.36	285	0.104	4.0		

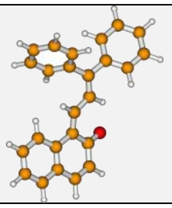	$S_0 \rightarrow S_6(n\pi^*)$	4.38	283	0.024	9.1		
**TT-H**	S_0	*0.62* [a]			*3.0* [b]		
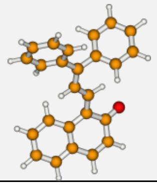	$S_0 \rightarrow S_1(\pi\pi^*)$	2.81	441	0.110	0.8		
	$S_0 \rightarrow S_2(\pi\pi^*)$	3.08	403	0.600	6.0	3.01 eV	412 nm
	$S_0 \rightarrow S_3(\pi\pi^*)$	3.63	342	0.052	7.5		
	$S_0 \rightarrow S_4(\pi\pi^*)$	4.20	295	0.149	5.9		
	$S_0 \rightarrow S_5(\pi\pi^*)$	4.35	285	0.098	4.7		
**AP-H**	S_0	*0.99* [a]			*1.4* [b]		
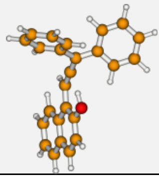	$S_0 \rightarrow S_1(\pi\pi^*)$	3.83	324	0.220	1.0		
	$S_0 \rightarrow S_2(\pi\pi^*)$	4.04	307	0.010	7.4		
	$S_0 \rightarrow S_3(\pi\pi^*)$	4.36	285	0.241	1.2		
	$S_0 \rightarrow S_4(\pi\pi^*)$	4.59	270	0.025	9.9		
	$S_0 \rightarrow S_5(\pi\pi^*)$	4.72	263	0.105	2.3		

[a] adiabatic energy of the given form (*Ea*, in eV) relative to the ground-state closed form, **CF**, calculated at the MP2/cc-pVDZ theory level. [b] dipole moment calculated at the MP2/cc-pVDZ theory level.

Table S3. Vertical excitation energy (ΔE^{VE}, in eV and λ_{abs}, in nm calculated *vs.* experimental) of the lowest singlet excited states for the equilibrium form of the respective singlet excited state minimum, [1]TC-OCH3, optimized at the ADC(2)/cc-pVDZ theory level. The UV-vis absorption spectra are calculated with the same method.

S_0 form		E^{VE} calc.	abs calc.	*f* calc.	E^{VE} expt.	abs expt.
**S_1-S_n vertical excitation energies at the singlet excited state TC minimum, [1]TC-OCH3**						
**S_1(TC)** 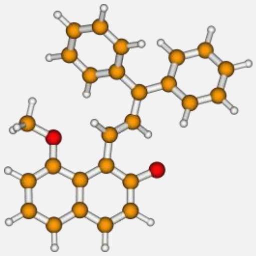	S_1	*2.15*[a]				
	$S_1 \rightarrow S_2$(n *)	0.62	2002	0.003		
	$S_1 \rightarrow S_3$(*)	2.17	572	0.013		
	$S_1 \rightarrow S_4$(*)	2.60	477	0.465	2.36	525 nm
	$S_1 \rightarrow S_5$(*)	3.10	400	0.025		
	$S_1 \rightarrow S_6$(n *)	3.26	381	0.006		
	$S_1 \rightarrow S_7$(*)	3.40	365	0.120		
	$S_1 \rightarrow S_8$(n *)	3.51	354	0.006		
	$S_1 \rightarrow S_9$(n *)	3.60	345	0.004		
	$S_1 \rightarrow S_{10}$(n *)	3.76	330	0.001		
	$S_1 \rightarrow S_{11}$(*)	3.78	328	0.012		
	$S_1 \rightarrow S_{12}$(n *)	3.81	326	0.005		
	$S_1 \rightarrow S_{13}$(*)	3.89	319	0.200	3.65	340 nm
	$S_1 \rightarrow S_{14}$(*)	3.94	315	0.022		
	$S_1 \rightarrow S_{15}$(n *)	4.01	309	0.005		

[a] adiabatic energy of the given form (*Ea*, in eV) relative to the ground-state closed form, **CF**, calculated at the MP2/cc-pVDZ theory level.

Table S4. Vertical excitation energy (ΔEVE, in eV and λabs, in nm calculated vs. experimental) of the lowest singlet excited states for the equilibrium form of the respective singlet excited state minimum, 1TC-H molecule, optimized at the ADC(2)/cc-pVDZ theory level. The UV-vis absorption spectra are calculated with the same method.

S_0 form		ΔE^{VE} calc.	λ_{abs} calc.	f calc.	ΔE^{VE} expt.	λ_{abs} expt.
**S_1-S_n vertical excitation energies at the singlet excited state TC minimum, ^{1}TC -H**						
	S_1	*2.14* [a]				
	$S_1 \rightarrow S_2(n\pi^*)$	0.67	1852	0.001		
	$S_1 \rightarrow S_3(\pi\pi^*)$	2.30	540	0.007		
**S_1(TC)**	$S_1 \rightarrow S_4(\pi\pi^*)$	2.60	477	0.440	2.39 eV	520 nm
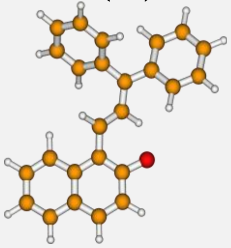	$S_1 \rightarrow S_5(\pi\pi^*)$	3.23	384	0.002		
	$S_1 \rightarrow S_6(\pi\pi^*)$	3.31	375	0.008		
	$S_1 \rightarrow S_7(\pi\pi^*)$	3.37	368	0.042		
	$S_1 \rightarrow S_8(\pi\pi^*)$	3.52	352	0.010		
	$S_1 \rightarrow S_9(\pi\pi^*)$	3.67	338	0.067		
	$S_1 \rightarrow S_{10}(\pi\pi^*)$	3.69	336	0.021		
	$S_1 \rightarrow S_{11}(\pi\pi^*)$	3.78	328	0.004		
	$S_1 \rightarrow S_{12}(\pi\pi^*)$	3.83	324	0.094	3.65	340
	$S_1 \rightarrow S_{13}(\pi\pi^*)$	3.89	319	0.067		
	$S_1 \rightarrow S_{14}(\pi\pi^*)$	3.91	317	0.082		
	$S_1 \rightarrow S_{15}(\pi\pi^*)$	3.99	311	0.003		

[a] adiabatic energy of the given form (*Ea*, in eV) relative to the ground-state closed form, **CF**, calculated at the MP2/cc-pVDZ theory level.

References

1. Kovalenko, S.A.; Schanz, R.; Farztdinov, V.M.; Hennig, H.; Ernsting, N.P., Femtosecond relaxation of photoexcited para-nitroaniline: solvation, charge transfer, internal conversion and cooling. *Chem. Phys. Lett.* **2000,** *323*, 312-322.
